# Longitudinal monitoring of *Ehrlichia ruminantium *infection in Gambian lambs and kids by pCS20 PCR and MAP1-B ELISA

**DOI:** 10.1186/1471-2334-7-85

**Published:** 2007-07-27

**Authors:** Bonto Faburay, Dirk Geysen, Susanne Munstermann, Lesley Bell-Sakyi, Frans Jongejan

**Affiliations:** 1 International Trypanotolerance Centre, PMB 14, Banjul, The Gambia; 2 Prince Leopold Institute of Tropical Medicine, Nationalestraat 155, B-2000 Antwerp, Belgium; 3 Centre for Tropical Veterinary Medicine, Royal (Dick) School of Veterinary Studies, University of Edinburgh, Easter Bush, Roslin, Midlothian EH25 9RG, UK; 4 Utrecht Centre for Tick-borne Diseases, Department of Infectious Diseases and Immunology, Faculty of Veterinary Medicine, Utrecht University, Yalelaan 1, 3584CL Utrecht, The Netherlands; 5 Department of Veterinary Tropical Diseases, Faculty of Veterinary Science, University of Pretoria, Private Bag X04, Onderstepoort 0110, South Africa

## Abstract

**Background:**

The epidemiology of *E. ruminantium *infection in extensively managed young animals is not adequately understood. Thus in this study, we monitored the onset (age at first infection) and kinetics of *E. ruminantium *infection and antibody response in extensively managed newborn lambs and kids at three sites in The Gambia.

**Methods:**

We used a nested pCS20 PCR and MAP1-B ELISA in a longitudinal study to monitor the onset (age at first infection) and kinetics of *E. ruminantium *infection and antibody response respectively, in 77 newborn lambs and kids under a traditional husbandry system at three sites (Kerr Seringe, Keneba, Bansang) in The Gambia where heartwater is known to occur. The animals were monitored for field tick infestation and the comparative performance of the two assays in detecting *E. ruminantium *infection was also assessed.

**Results:**

The infection rate detected by pCS20 PCR varied between 8.6% and 54.8% over the 162-day study period. Nineteen per cent of the animals in week 1 post-partum tested positive by pCS20 PCR with half of these infections (7/14) detected in the first 3 days after birth, suggesting that transmission other than by tick feeding had played a role. The earliest detectable *A. variegatum *infestation in the animals occurred in week 16 after birth. Antibodies detected by MAP1-B ELISA also varied, between 11.5% and 90%. Although there is considerable evidence that this assay can detect false positives and due to this and other reasons serology is not a reliable predictor of infection at least for heartwater. In contrast to the pCS20 PCR, the serological assay detected the highest proportion of positive animals in week 1 with a gradual decline in seropositivity with increasing age. The pCS20 PCR detected higher *E. ruminantium *prevalence in the animals with increasing age and both the Spearman's rank test (*r*_*s *_= -0.1512; P = 0.003) and *kappa *statistic (-0.091 to 0.223) showed a low degree of agreement between the two assays.

**Conclusion:**

The use of pCS20 PCR supported by transmission studies and clinical data could provide more accurate information on heartwater epidemiology in endemic areas and single-occasion testing of an animal may not reveal its true infection status. The view is supported because both the vector and vertical transmission may play a vital role in the epidemiology of heartwater in young small ruminants; the age range of 4 and 12 weeks corresponds to the period of increased susceptibility to heartwater in traditionally managed small ruminants.

## Background

Heartwater is an infectious disease of ruminants caused by a rickettsia, *Ehrlichia ruminantium*, and transmitted by ixodid ticks of the genus *Amblyomma*. The disease is endemic in sub-Saharan Africa and on some islands in the Caribbean.

The epidemiology of heartwater in young small ruminants is not adequately understood. In heartwater-endemic areas where extensive husbandry systems exist and tick control is absent or limited, the numbers of *Amblyomma *ticks are high and animals are subjected to almost continuous tick, and presumably *E. ruminantium *challenge [1]. Several researchers postulated that the existence of endemic stability for *E. ruminantium *and tick-borne infections in general may be dependent on infection, by tick transmission, to the very young host during a period of reduced susceptibility to clinical disease [2-4]. It has been reported that newborn calves, lambs and kids possess an inverse age-related resistance to heartwater, which is independent of the immune status of the dam[5,6]; this resistance has been reported to be of short duration, lasting 9 days in lambs [7] and 2 weeks in kids [8]. However, the concept of endemic stability in relation to heartwater in extensively managed small ruminants in The Gambia (local dwarf sheep and goats) is not completely understood and may not be the same as in the case of indigenous cattle. Mortality due to heartwater has been reported frequently in the first two species; and a 12-month risk assessment in The Gambia showed that indigenous small ruminants (local dwarf sheep and dwarf goats) experienced a much lower *A. variegatum *tick attachment rate of 0.76 ticks/animal than N'Dama cattle (9.36 ticks/animal) (B. Faburay et al., unpublished data). Moreover, evidence has been provided of possible occurrence of vertical transmission of *E. ruminantium *in calves [9] and that initial transmission of heartwater to calves may not always be by the tick vector [10], findings which could also apply to small ruminants.

Diagnostic tests targeting pCS20 sequences have long been considered specific for *E. ruminantium *[11,12] and recent advances in molecular diagnostics resulted in the development of a specific and sensitive pCS20 polymerase chain reaction (PCR) assay for detection of all known strains of *E. ruminantium *in ticks [11,13]. Previous experiments showed that the pCS20 PCR could detect *E. ruminantium *carrier infections in animals [14]. Preliminary random testing of suspected carrier small ruminants in a heartwater-endemic area (Keneba) in The Gambia using a nested pCS20 PCR detected a 60% *(n *= 14) infection rate; moreover all samples collected from clinically sick goats maintained on-station at ITC (Kerr Seringe), and confirmed as heartwater cases post mortem, tested positive by the same assay (B. Faburay, unpublished data). In the past, serological tests for detection of antibodies to *E. ruminantium *suffered from poor specificity due to cross-reactions with other ehrlichial agents [15-17]. Although the MAP1-B ELISA [17] has been reported to detect false-positives in heartwater-free areas attributed to cross-reactions with closely related species [18,19], the assay has higher sensitivity compared to other serological tests to detect *E. ruminantium *antibodies in ovine and caprine sera [20,21]. This increased sensitivity is attributed to the comparatively longer persistence of MAP1 antibodies in these species [17,20]. In the present study, we monitored the onset (age at first infection) and kinetics of *E. ruminantium *infection and antibody response in extensively managed newborn lambs and kids at three sites in The Gambia where heartwater is known to occur [22] using pCS20 PCR, and also compared the performance of the pCS20 PCR and indirect MAP1-B ELISA in detecting heartwater infection in small ruminants.

## Methods

### Study sites

Seventy-seven small ruminants (local dwarf sheep and dwarf goats) were monitored from birth for up to 162 days at three sites in The Gambia: Kerr Seringe (13°43' N, 16°72' W), Keneba (13°20' N, 16°01' W) and Bansang (13°27' W, 14°40' N). The animals at Kerr Seringe and Keneba belonged to the International Trypanotolerance Centre (ITC), whereas those in Bansang were animals of local smallholders. A recent serological study showed presence of MAP1-B specific antibodies at all three sites [22]. In the presence of *A. variegatum *ticks in these areas [23], we presume that these antibodies are due to exposure to *E. ruminantium*. Kerr Seringe is located in the western part of The Gambia on the coast, whereas Keneba is about 150 km east of Kerr Seringe. Bansang is located 150 km further east from Keneba. Monitoring of death in extensively managed small ruminants in Kerr Seringe from 1996 to 1999 showed that 17.9% of deaths in local dwarf sheep and 12.5% in local dwarf goats were associated with heartwater; and in Keneba these figures were 36% and 25% for sheep and goats respectively [24]. Although cases of mortality due to hearwater have been observed in sheep and goats in Bansang area, records of these deaths were not kept. *E. ruminantium *tick infection rates at these sites have been described previously [23].

### Animals and husbandry system

The study animals comprised 29 lambs at Kerr Seringe, 21 kids and 13 lambs at Keneba and 10 kids and 4 lambs at Bansang. All newborn animals and their dams were maintained under a traditional husbandry system without acaricide treatment, except for the dams at Kerr Seringe, which had received regular monthly acaricide treatment for about 10 months before the start of this study. Acaricide treatment of all animals was discontinued during the study period. All newborn lambs and kids, together with their dams, were maintained under the traditional husbandry system [25] without acaricide treatment. Under this system, animals were allowed to wander freely around the homestead for feed and food residues and/or graze in the bush during the day. Newborn kids and lambs were mostly tethered until they reached the age of 2 to 3 weeks of age to gain strength to graze with their dams in the bush. At night, depending on the site, the animals were usually penned in sheds or barns.

### Sampling and vector dynamics

Sampling and monitoring started in mid-February 2002, following the peak incidence of *A. variegatum *nymph attachment [26], and ended in mid-July 2002, the beginning of peak abundance of the adult instars. At the study sites, peak activity of *Amblyomma *nymphs occurs from November to January, which is followed by rapid decline or virtual disappearance of the tick population (both nymphs and adults) until May/June when adult ticks start to appear on animals [27], most frequently on cattle. Whole blood was collected with and without anticoagulant (EDTA) from each newborn animal on the day of birth or at the latest within 3–10 days of birth, and thereafter weekly and fortnightly for testing by pCS20 PCR and MAP1-B ELISA respectively. All newborn animals (0–10 days of age) were examined for ticks at the time of first sampling. Thereafter, selected animals (5 kids and 5 lambs at Keneba, 5 lambs at Kerr Seringe and all 16 neonates at Bansang) were examined weekly for tick infestation by examining the whole body. Post mortem examinations were carried out on all animals that died; and Giemsa-stained brain smears were examined for the presence of *E. ruminantium *inclusions in brain capillary endothelial cells [28]. The pCS20 PCR is the method of choice for detection of *E. ruminantium *[29] and analysis by nested pCS20 PCR assay as described below was carried out on the brains of animals, which showed hyperthermia and suspicious clinical symptoms characteristic of heartwater prior to death but were negative microscopically.

### DNA extraction

Blood with EDTA was introduced into plain glass capillary tubes and centrifuged for 5 minutes at 14 000 × *g *to separate the buffycoat using Hawksley^® ^HaematoSpin 1400. The tubes were broken and the buffycoats were applied to Whatman^® ^filter paper #3 or #4 and allowed to dry at room temperature. DNA was extracted by the Modified Plowe extraction method [30]. DNA from brain tissues of dead animals was extracted using the protocol for purification of total DNA from animal tissues in DNeasy^® ^Blood and Tissue Handbook (QIAGEN, Hilden Germany).

### pCS20 PCR analysis

Previous experiments already showed the specificity of the pCS20 PCR assay for *E. ruminantium *using AB128 and AB129 primers [12,13]. A nested PCR was carried out as described previously [23]. Briefly, AB128 and AB129 primers [13] were used as internal primers. AB129 was also used as the external reverse primer, while ITM130 (5' TCAATTGCTTAATGAAGCACTAACTCAC 3') was used as the new external forward primer. PCR amplification was carried out in a 25 μl volume comprising 5 μl DNA sample, 50 mM KCl, 10 mM Tris-HCl (pH 8.3), 1.65 mM MgCl_2_, 400 pmol of each of the deoxynucleoside triphosphates, dATP, dCTP, dGTP and dTTP, 0.4 U of Taq polymerase, 40 pmol of each primer. After a denaturing step of DNA at 94°C for 3 min, the first round of amplification was carried out using the following conditions: 39 cycles of 30 sec denaturation at 94°C, 45 sec annealing at 62°C and 1 min elongation at 72°C and a final extension of 10 min at 72°C. Aliquots of 0.5 μl of PCR product from the first round amplification were transferred as template to a second round of PCR at 84°C (hot start principle). The second round consisted of 25 cycles of the same PCR conditions as in the first round except for the annealing temperature, which was set at 58°C. In each PCR run, positive and negative controls were included. Positive controls were derived from *E. ruminantium *(Senegal isolate) DNA obtained from cell culture-derived organisms and negative controls were reagent blank samples without DNA. The PCR amplified a 279 bp fragment of open reading frame 2 of the 1,306-bp pCS20 sequence. Amplicons were separated by 1.5% agarose gel electrophoresis, stained with ethidium bromide, and visualized under ultraviolet light.

### Indirect MAP1-B ELISA

Serum was separated by centrifugation from the blood samples collected without anticoagulant, and stored at -20°C until required. The *E. ruminantium *MAP1-B ELISA was carried out as described previously [17,31]. The assay has been shown to have high sensitivity for detection of *E. ruminantium *antibodies [32] particularly in small ruminants [18,20,31,33] with a specificity of 98.9% and 99.4% for caprine and ovine sera respectively [17,34]. Each serum sample was tested in duplicate. For sheep, each test included duplicate positive control sera obtained from a heartwater-naïve sheep of the Tesselaar breed experimentally infected with the Senegal isolate of *E. ruminantium *[35] at the Faculty of Veterinary Medicine, Utrecht, The Netherlands. Duplicate negative control sera were obtained from the same sheep prior to infection. Similar controls were obtained from a Saanen goat and included in duplicate for testing goat sera. Species-specific second step IgG antibodies conjugated with horseradish peroxidase (Nordic Immunology, Tilburg – The Netherlands) were used. Optical densities of the ELISA tests were measured using a Titertek Multiskan^® ^ELISA reader (Titertek, Flow Laboratories Inc.) at 405 nm wavelength. For each plate, the cut-off value was calculated as two times the percentage positivity of the negative control serum relative to the positive control serum [14,17].

### Statistical analysis

For analysis of data, the PCR and ELISA test results of the sample animals were grouped according to age: 0 – 10 days, 11 – 21 days, 22 – 49 days, 50 – 77 days, 78 – 98 days, 99 – 126 days, and 127 – 162 days. Correlation analysis was carried out using Spearman's rank correlation coefficient (*r*_*s*_) of Stata^® ^statistical programme to assess the overall level of association between the pCS20 PCR and the MAP1-B ELISA in monitoring the kinetics of *E. ruminantium *infection in lambs and kids. Proportion of animals positive for *E. ruminantium *infection determined by both assays was deduced using the same statistical programme. Agreement between the two assays in detection of *E. ruminantium *infection in animals of the various age categories was assessed by method of determining the *kappa *statistic:

*kappa *= (*OP *- *EP*)/(1 - *EP*),

where *OP *= (*a *+ *d*)/*n*; the observed proportion agreement between the two tests

*n *= (*a *+ *b *+ *c *+ *d*)

*EP *= [{(*a *+ *b*)/*n*} × {(*a *+ *c*)/*n*}] + [{(*c *+ *d*)/*n*} × {(*b *+ *d*)/*n*}]; the expected proportion of agreement by chance

*Kappa *ranges from 1 (complete agreement) to 0 (agreement is equal to that expected by chance), whereas negative values indicate agreement less than is expected by chance. Benchmarks for interpreting *kappa *values were defined according to Everitt [36]:

>0.81: almost perfect agreement; 0.61 – 0.80: substantial agreement; 0.41 – 0.61:moderate agreement; 0.21 – 0.40: fair agreement; 0 – 0.20: slight agreement; 0: poor agreement

The method of Kaplan-Meier survival estimate was used for survival analysis.

## Results

### Tick counts

Results of total tick counts carried out weekly on animals at each of the three sample sites are shown in Table 1. The highest level of *A. variegatum *infestation was recorded in animals at Keneba, with a total of 40 ticks, consisting of 2 nymphs and 38 adult ticks, detected throughout the observation period. Lower levels of *A. variegatum *infestation were recorded at Kerr Seringe and Bansang, with 1 nymph and 2 female *A. variegatum *ticks detected respectively. Amongst the young lambs and kids that died of heartwater, *A. variegatum *ticks were recorded on 1 animal (#1317) at Kerr Keringe and 3 animals, #4333, #4338 and #4340, at Keneba (Table 2 and 3). Generally, tick infestations occurred at the end of the observation period in the early rainy season (mid-June and July); and infestation was first detected on animals at week 16 after birth.

**Table 1:** Weekly *A. variegatum *tick counts on lambs and kids examined at the three sample sites in The Gambia

**Week of observation**	**Total no. of *A. variegatum *ticks counted on animals at**		
	
	**Kerr Seringe **(5 lambs)	**Keneba **(5 lambs, 5 kids)	**Bansang **(5 lambs, 11 kids)
1	0	0	0
2	0	0	0
3	0	0	0
4	0	0	0
5	0	0	0
6	0	0	0
7	0	0	0
8	0	0	0
9	0	0	0
10	0	0	0
11	0	0	0
12	0	0	0
13	0	0	0
14	0	0	0
15	0	0	0
16	0*	1N, 1M	0
17	0	0	0
18	1N	7M, 4F	2F**
19	0	4M	0
20	0	14M, 4F	0
21	0	0	0
22	0	0	0
23	0	1M, 1F	0

* 2 *Hyalomma *sp. adults and 1 *Rhipicephalus *sp. adult also recorded

** 1 *Hyalomma *sp. adult also recorded

*Key: *N = nymph, M = adult male, F = adult female

**Table 2:** Periods of detection of *E. ruminantium *DNA and antibodies in lambs at various age levels sampled in Kerr Seringe

**Animal No**.	**^1^Sp**.	**^2^Period DNA detected (PCR)**							**Period antibodies detected (MAP1-B)**							**Status**	**^5^*AV***
		
		**1**	**2**	**3**	**4**	**5**	**6**	**7**	**1**	**2**	**3**	**4**	**5**	**6**	**7**	Survived	
1301	L	-	-	-	-	+	nd	+	+	+	+	-	-	-	-	Survived	0
1302	L	-	-	-	-	+	nd	+	+	+	+	-	-	-	-	Survived	
1303	L	-	-	+	-	+	nd	-	+	+	+	-	-	-	-	Survived	0
1304	L	-	-	-	-	-	nd	-	+	+	-	-	-	-	-	Survived	
1305	L	-	-	+	+	-	nd	+	+	+	+	-	-	-	-	Survived	
1308	L	-	-	-	+	+	nd	-	+	+	+	+	-	-	-	Survived	
1309	L	-	-	+	+	+	nd	-	+	+	+	-	-	-	-	Survived	
1311	L	-	-	+	-	-	nd	-	+	+	+	+	+	+	+	Survived	
1312	L	-	-	-	-	-	nd	-	+	+	+	+	-	-	-	Survived	
1313	L	-	-	+	-	+	nd	-	+	+	+	-	-	-	-	Survived	
1314	L	-	-	-	-	-	nd	-	+	-	-	-	-	-	-	Survived	0
1315	L	-	+	-	-	-	nd	-	+	nd	+	+	-	-	-	Survived	
1316	L	-	-	-	-	-	nd	+	+	-	-	-	-	-	-	Survived	
1318	L	-	-	-	nd	+	nd	+	+	+	+	-	-	-	-	Survived	
1319	L	-	-	-	-	-	nd	-	+	+	+	+	-	-	-	Survived	0
1320	L	-	-	+	+	+	nd	+	+	nd	+	+	-	-	-	Survived	
1322	L	-	-	-	nd	+	nd	-	+	+	+	+	-	-	-	Survived	
1323	L	-	-	-	nd	+	nd	-	+	+	+	+	+	+	-	Survived	
1325	L	-	-	-	nd	-	nd	-	-	-	-	-	-	-	-	Survived	
1327	L	-	-	-	-	-	nd	-	+	+	+	+	-	-	-	Survived	
1328	L	-	-	-	nd	-	nd	+	+	+	+	+	-	-	-	Survived	
1329	L	-	-	-	nd	-	nd	-	-	-	-	+	-	-	-	Survived	
1330	L	-	-	-	-	nd	-	nd	+	+	+	+	-	-	-	Survived	
1331	L	-	-	-	-	nd	-	nd	+	+	+	+	-	-	nd	Survived	
**1333^6^**	L	nd	+						+	+						^3^D(28) ^4^hw-G	
**1332**	L	-	-	-					+	+	+					D(42*)	
**1321**	L	-	-	-					+	+	+					D(42*)	
**1306**	L	-	-	-	-				+	+	-	-				D(77*)	
**1317**	L	-	-	-	nd				+	+	+	+				D(84) hw-G	1 N

***Key***: ^1^Sp. = Species; L = lamb; K = kid; ^2^Age range per level: **1 **(0 – 10 days), **2 **(11–21 days), **3 **(22 – 49 days), **4 **(50 – 77 days), **5 **(78 – 98 days), **6 **(99 – 126), **7 **(127 – 162); ^3^D = died (numbers in parentheses indicate day of death); ^4^hw-G heartwater confirmed by Giemsa; hw-PCR: heartwater confirmed by pCS20 PCR;

^5^*AV: A. variegatum *tick counts on selected study animals; N = nymph; A = adult; *mortality due to coccidial and/or clostridial enteritis; ^6^Animal numbers in bold are cases of mortality

**Table 3:** Periods of detection of *E. ruminantium *DNA and antibodies in all lambs and kids at various age levels sampled in Keneba

**Animal No**.	**Sp**.	**Week DNA detected (PCR)**							**Week antibodies detected (MAP1-B)**							**Status**	***AV***
		
		**1**	**2**	**3**	**4**	**5**	**6**	**7**	**1**	**2**	**3**	**4**	**5**	**6**	**7**		
4308	K	-	-	-	-	-	nd	+	+	+	+	-	-	-	-	Survived	1 N
4309	K	+	-	-	-	-	nd	+	nd	-	-	-	-	-	-	Survived	
4312	K	+	-	+	-	-	nd	+	+	+	+	+	-	+	-	Survived	5A
4314	K	-	-	+	-	-	nd	-	+	+	+	-	-	-	-	Survived	
4315	K	-	+	+	-	-	nd	+	+	+	+	-	-	+	-	Survived	
4318	K	+	-	+	-	-	-	-	+	+	-	-	-	-	-	Survived	
4319	K	+	-	+	-	-	+	-	+	+	-	-	-	-	-	Survived	
4325	K	+	-	+	+	+	+	+	+	+	-	-	-	-	-	Survived	
4331	K	-	nd	-	-	-	-	-	+	-	-	-	-	-	-	Survived	
4332	K	-	nd	+	-	-	-	-	+	+	+	+	-	-	-	Survived	
4335	K	-	-	-	+	-	+	-	nd	-	+	-	-	-	-	Survived	
4336	K	-	-	+	+	+	+	-	+	+	-	-	-	-	-	Survived	
4372	K	nd	-	-	+	+	-	-	nd	+	+	-	+	+	+	Survived	6A
4376	K	-	nd	+	-	-	-	-	+	+	+	+	+	+	-	Survived	
4377	K	-	nd	-	-	-	nd	-	+	+	+	-	-	-	-	Survived	
4378	K	-	nd	-	-	+	nd	-	+	+	+	+	-	-	-	Survived	
4306	L	nd	-	+	-	nd	+	+	nd	+	+	+	+	-	+	Survived	8A
4311	L	-	-	-	-	-	nd	+	+	+	+	+	-	-	+	Survived	
4316	L	+	-	+	-	+	nd	+	+	+	-	+	-	-	+	Survived	
4321	L	+	+	+	+	+	+	-	+	+	+	+	-	+	-	Survived	7A
4323	L	+	-	+	+	+	+	-	+	-	-	+	-	-	+	Survived	
4324	L	+	-	nd	+	+	-	+	+	+	+	+	+	-	-	Survived	
4326	L	-	nd	-	-	-	-	-	nd	+	-	+	+	-	-	Survived	
4337	L	+	-	-	-	+	+	-	+	+	+	-	-	+	-	Survived	4A
**4339**	L	+	-	-					-	-	-					D(42) hw-G	
**4375**	K	-	-	-					+	+	+					D (42*)	
**4333**	L	+	+	+					+	-	+					D(56) hw-G	1 N, 3A
**4379**	K	-	nd	-	-				+	nd	-	-				day 56*	
**4340**	K	-	-	-	+				-	-	-	-				D(63) hw-PCR	2A
**4338**	L	-	-	+	-				+	+	+	+				D(63) hw-G	3A
**4320**	K	-	-	-	+				nd	+	+	-				D(91) hw-PCR	
**4334**	L	-	-	-	-	+			+	+	+	+	+			D(112) hw-PCR	
**4305**	L	nd	-	-	-	nd	+		nd	+	+	+	+	-		D(147) hw-G	
**4313**	kid	+	-	-	-	-	+	+	+	+	-	-	-	-	-	day 147*	

### pCS20 PCR and indirect MAP1-B ELISA

The pCS20 PCR assay detected *E. ruminantium *DNA in 57 out of the 77 animals at least once during the study period. Fifteen animals (excluding those that died), 9 in Kerr Seringe, 3 in Keneba and 3 in Bansang, remained negative by this test throughout the study (Table 2, 3 and 4). Data for a number of animals at different age levels were unavailable. Batches of some buffycoat extracts on filter paper (for DNA) stored in the freezer were soaked with water due to a freezer failure rendering some samples unsuitable for further analysis; the batch of samples of level 6 in Kerr Seringe was particularly affected (Table 2). Absence of serological data for some animals at different time periods was mainly due to erasure of ink labels on the serum cryotubes during handling and storage making them unidentifiable. Such samples were excluded from further analysis. The highest number of PCR-positive animals was observed at 78 to 98 days of age. Of the 9 animals (6 kids and 4 lambs) that were sampled immediately after birth (0–3 days) at Keneba, 6 tested positive by PCR; at Bansang, 1 animal was positive, whereas none tested positive at Kerr Kerr Seringe (Table 5). Nineteen per cent of the animals sampled after birth (from day 0 to 10 days), tested positive by PCR (Table 6). The serological assay detected the highest proportion of positive small ruminants (90%) at week 1 of age (0 – 10 days). A decline in the number of serologically positive animals was observed with increasing age (Table 6). The performance of the pCS20 PCR and the MAP1-B ELISA tests in detecting *E. ruminantium *in lambs and kids of the different age categories are outlined in Tables 2, 3, 4 and 5. All 7 neonates, between 0 and 3 days age range detected positive by PCR, were correspondingly serologically positive (Table 5). Four of these animals (4321, 4323, 4324 and 338) remained intermittently PCR and MAP1-B positive in subsequent weeks (Table 3 and 4) suggesting persistence of infection. *E. ruminantium *antibodies could not be detected in three PCR-positive neonates (4318, 4319 and 4325) after the first two weeks until the end of the study (Table 3). Assessment of agreement between the two assays by method of determining *kappa *statistic is shown in Table 7. The *kappa *coefficient ranged from -0.091, indicating agreement less than is expected by chance, to 0.223, suggesting fair agreement, between the two assays (Table 7). Global comparison of the results by Spearman's rank test showed that the two assays did not always agree (*r*_*s *_= -0.1512; *P *= 0.003) and data (Table 2, 3, 4 and Table 6) showed that the pCS20 PCR detected higher *E. ruminantium *infection rates with increasing age in field-exposed small ruminants. For example, a number of animals (#1301, #1302, #1316, #1318, #4325, #2317 and #2318) that tested PCR-positive at 99 to 162 days of age were correspondingly (the same age interval) negative by MAP1-B ELISA. The serology results showed that most neonates tested between 0–3 days of age carried antibodies to MAP1-B antigen of *E. ruminantium *(Table 5).

**Table 4:** Periods of detection of *E. ruminantium *DNA and antibodies in all lambs and kids at various age levels sampled in Bansang

**Animal No**.	**Sp**.	**Week DNA detected (PCR)**							**Week antibodies detected (MAP1-B)**							**Status**	***AV***
		
		**1**	**2**	**3**	**4**	**5**	**6**	**7**	**1**	**2**	**3**	**4**	**5**	**6**	**7**		
330	K	-	-	-	-	+	-	+	-	-	-	nd	+	-	-	Survived	0
336	K	-	-	+	-	+	-	+	nd	+	+	+	nd	+	-	Survived	0
338	K	+	-	-	-	+	+	+	nd	+	-	+	nd	+	-	Survived	0
374	K	-	-	-	-	+	nd	+	+	+	+	-	+	-	-	Survived	0
2313	K	-	-	-	-	+	+	-	nd	nd	nd	nd	nd	-	+	Survived	0
2314	K	-	-	-	-	+	+	+	+	+	+	+	nd	-	-	Survived	0
2315	K	-	-	-	+	+	+	-	nd	nd	nd	nd	nd	+	-	Survived	0
2316	K	-	+	-	+	-	nd	-	nd	nd	nd	-	-	-	-	Survived	1A
2317	K	-	-	-	-	nd	+	+	nd	nd	nd	+	-	-	-	Survived	0
2318	K	-	-	-	+	nd	+	+	nd	nd	nd	+	-	-	-	Survived	0
2351	L	-	-	-	-	-	-	-	+	+	+	+	-	-	-	Survived	0
2352	L	-	-	-	-	-	-	-	+	-	+	-	-	-	nd	Survived	1 A
2353	L	-	nd	-	-	-	-	nd	-	-	-	nd	-	-	-	Survived	0
2354	L	-	-	-	-	+	+	nd	-	-	-	+	+	-	-	Survived	0

**Table 5:** Outcome of pCS20 PCR and MAP1-B ELISA tests on samples collected from animals at 0 – 3 days after birth

**Anim. No**.	**Species**	**Site**	**Day of first sample**	**PCR**	**MAPI-B**
305	lamb	Kerr Seringe	1	-	+
1306*	lamb	Kerr Seringe	1	-	+
1313	lamb	Kerr Seringe	0	-	+
1315	lamb	Kerr Seringe	3	-	+
1318	lamb	Kerr Seringe	2	-	+
4318	kid	Keneba	0	+	+
4319	kid	Keneba	0	+	+
4325	kid	Keneba	3	+	+
4331	kid	Keneba	2	-	+
4332	kid	Keneba	1	-	+
4335	kid	Keneba	2	-	nd
4321	lamb	Keneba	0	+	+
4323	lamb	Keneba	3	+	+
4324	lamb	Keneba	1	+	+
330	kids	Bansang	3	-	-
336	kids	Bansang	3	-	nd
338	kids	Bansang	1	+	nd
374	kids	Bansang	0	-	+
2313	kids	Bansang	3	-	nd
2314	kids	Bansang	3	-	+
2315	kids	Bansang	0	-	nd
2316	kids	Bansang	3	-	nd
2317	kids	Bansang	1	-	nd
2318	kids	Bansang	3	-	nd
2351	lamb	Bansang	2	-	+
2352	lamb	Bansang	1	-	+
2353	lamb	Bansang	3	-	-
2354	lamb	Bansang	3	-	-

*the only mortality (occurred on day 77) among the group and was due to clostridial enteritis

**Table 6:** Prevalence of *E. ruminantium *DNA and antibodies in lambs and kids according to age as determined by pCS20 PCR and MAP1-B ELISA

**Age group**	**pCS20 PCR **percent % (*n*)			**MAP1-B ELISA **percent % (*n*)		
	
	Lambs	Kids	Overall	Lambs	Kids	Overall
0 – 10 days	16.3 (43)	23.3 (30)	19.2 (73)	88.3 (43)	90.0 (20)	90.0 (63)
11 – 21 days	8.9 (45)	8.3 (24)	8.6 (69)	77.3 (44)	80.0 (25)	78.3 (69)
22 – 49 days	27.3 (44)	32.3 (31)	29.3 (75)	75.6 (45)	50.0 (26)	66.2 (71)
50 – 77 days	20.6 (34)	30.0 (30)	25.0 (64)	65.0 (40)	33.3 (27)	52.2 (67)
78 – 98 days	48.6 (35)	40.0 (25)	45.0 (60)	18.4 (38)	22.7 (22)	20.0 (60)
99 – 126 days	42.9 (14)	64.7 (17)	54.8 (31)	10.8 (37)	25.9 (27)	17.2 (64)
127 – 162 days	34.3 (32)	48.1 (27)	40.8 (59)	14.7 (34)	7.4 (27)	11.5 (61)

Number of samples tested per age group in parentheses

**Table 7:** Number of lambs and kids tested for *E. ruminantium *(ER) infection by pCS20 PCR and MAP1-B ELISA at the various age levels and the values of kappa statistic

**Age Group**	**pCS20 PCR**	**MAP1-B ELISA**		***Kappa***
			
		ER positive	ER negative	
0 – 10 days	ER positive	11(*a*)	1(*b*)	0.015
	ER negative	44(*c*)	6(*d*)	
11 – 21 days	ER positive	3	1	-0.015
	ER negative	46	8	
22 – 49 days	ER positive	17	6	0.068
	ER negative	30	16	
50 – 77 days	ER positive	6	8	-0.019
	ER negative	24	19	
78 – 98 days	ER positive	7	16	0.223
	ER negative	3	28	
99 – 126 days	ER positive	4	12	0.035
	ER negative	3	11	
127 – 162 days	ER positive	3	21	0.009
	ER negative	4	30	

### Mortality

**Figure 1:**
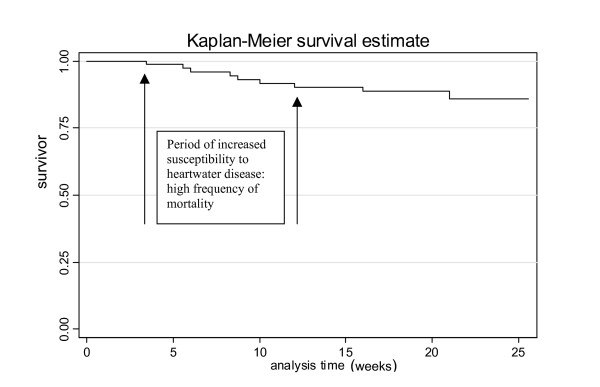
Heartwater mortality data: Kaplan-Meier survival estimate.

Fifteen animals (10 lambs and 5 kids) died during the course of the study, between 28 and 147 days (Table 2, 3 and 4), representing a crude mortality rate of 19.5% (15/77); nine deaths (Figure 1; Table 2, 3 and 4) were confirmed heartwater-associated by detection of *E. ruminantium *in brain-crushed smears (6 deaths) or detection of *E. ruminantium*-specific pCS20 sequences in brain tissue DNA extracts (3 deaths). The 3 deaths confirmed by PCR occurred in Keneba and the lack of Giemsa-stained brain-positive results was attributed to poor storage of the brains prior to shipment to the laboratory for analysis. The three animals, however, showed clinical symptoms (hyperthermia, nervous symptoms) prior to death, and clinical pathological changes (hydropericardium, hydrothorax) at post mortem, which although not pathognomonic but are characteristic for heartwater. Overall case mortality rate for the 3 sites was 11.7% (9/77). At Kerr Seringe, the crude mortality rate was 17.2% (5/29) and case (heartwater) mortality rate was 6.9% (2/29); Keneba showed a crude mortality rate of 29.4% (10/34) and a higher case mortality rate of 20.6% (7/34), which could be associated with the high tick attack rate recorded at this site; in contrast to Bansang, no mortality occurred amongst the animals during the study period (Table 2, 3 and 4). Six animals were negative for *E. ruminantium*, and death was attributed mainly to coccidial or clostridial enteritis. Of the 9 animals which died of heartwater, 6 were previously positive for *E. ruminantium *by both PCR and MAP1-B ELISA. Five of the 9 animals that died of heartwater (#4305, #4334, #4338, #4320 and #4340) showed a first positive PCR test after 21 days of age (Table 2, 3 and 4) outside the brief period of inverse age-related resistance. Survival analysis (Figure 1) showed the frequency of mortality due to heartwater among extensively managed small ruminants to be higher in animals aged between 4 and 12 weeks.

## Discussion

The present study used pCS20 PCR to monitor the onset (age at first infection) and kinetics of *E. ruminantium *infection in neonatal lambs and kids maintained under a traditional husbandry system in three major locations of livestock production in The Gambia where heartwater is known to occur. It also compared the performance of the pCS20 PCR and indirect MAP1-B ELISA in detecting *E. ruminantium *infections in small ruminants. The prevalence of infection detected by pCS20 PCR varied between 8.6% and 54.8% over the 162-day study period. Nineteen per cent (14/73) of the animals in week 1 post-partum (0–10 days of age) were positive by pCS20 PCR. Half of these infections (7/14) were detected in the first 3 days of life (Table 5), suggesting that transmission other than by tick feeding had played a role. In this study, the earliest detectable *A. variegatum *infestation occurred in week 16 (Table 1). A plausible explanation of this finding is that vertical transmission of *E. ruminantium *from the dam to the offspring could possibly be occurring in small ruminants maintained under the traditional husbandry system. Vertical transmission was demonstrated to occur in cattle in Zimbabwe under natural field conditions [9], although the mode of transmission, either *in utero *or with colostrum, remains to be elucidated. Many tick-borne pathogens related to *E. ruminantium*, such as *Anaplasma (Ehrlichia) phagocytophilum *[37], *E. risticii *[38], *Anaplasma *spp. [39] and *Coxiella burnetti *[40] can be transmitted *in utero*. Interestingly, in our study, most of the neonatal infections detected by pCS20 PCR occurred in animals of 0 to 3 days of age and were located in Keneba (67%, 6/9; Table 5) where there is a comparatively high *A. variegatum *tick abundance [23] and minimal or no tick control was practised. The traditional management system of small ruminants in The Gambia substantially diminishes the likelihood of tick infestation of neonates at or immediately after birth. Parturitions at Keneba and Kerr Seringe occurred in pens, which were regularly cleaned; and at Keneba all births happened precisely between 11 March and 8 April, coinciding with the period of significant decline in *Amblyomma *nymphs (or near complete disappearance) and least activity of *Amblyomma *ticks [27]. Furthermore, the period required for transmission of *E. ruminantium *to occur after attachment of an infected tick to a susceptible host is estimated between 27 and 38 hours for nymphs and between 51 and 75 hours for adults [41], and it is highly unlikely that *E. ruminantium *transmitted to neonates at birth through tick bite could appear in the blood stream on the very day of or the following day after birth and detected by PCR. Against this background, it is postulated that vertical transmission of *E. ruminantium *possibly occurs in traditionally managed small ruminants and there is increased likelihood of this occurrence under conditions of medium to high tick challenge in the absence of, or with minimal, tick control. Interestingly, based on the number of PCR-positives, the phenomenon appeared to be more evident in Keneba (Table 5), a site located in the western part of The Gambia characterized by comparatively high rates of *E. ruminantium *tick infection [23] and tick attachment [22]. Bansang, which is in the eastern part of the country with low rates of tick infection and tick attachment [22,23], showed only 1 positive case. At Kerr Seringe, none of the neonates tested positive (Table 5). The latter site is also located in the western part of the country characterized by high rates of tick infection and tick attachment; and the absence of positive tests in the neonates was attributed their dams being subjected to regular treatment with acaricide as described above. Intensive use of acarides in indigenous livestock results in disruption of endemic stability to tick-borne diseases through disruption of infection by tick transmission [42-45]. It was highly likely that regular acaricide treatment of dams at Kerr Seringe resulted in significant reduction of infection intensity that was required for transmission of infection from dam to offspring to occur. Alternatively, the pCS20 PCR could be detecting *E. ruminantium *of low pathogenicity or an as yet uncharacterized related organism in these animals, which does not cause disease [46] and is transmitted *in utero*, neonatally, or by some other route. It should be mentioned that the present study was not designed to demonstrate the occurrence of vertical transmission of *E. ruminantium *in traditionally managed small ruminants but the findings herein strongly suggested plausibility of the phenomenon and should stimulate further investigation and confirmation through tick feeding experiments. Additionally, it was observed that a significant number of animals tested negative in day-11 to 21-age range by PCR and later became positive in day 22 to 49, with some animals demonstrating intermittent positivity throughout the study period suggesting that the level of *E. ruminantium *rickettsaemia in the peripheral blood fluctuated and sometimes it was not possible to detect infection [29].

Presuming that heartwater infections were the cause of the sero-positive reactions, prevalence of *E. ruminantium *infection detected by MAP1-B ELISA also varied, between 11.5% and 90%. However, serological assays, generally, unlike PCR, do not show infection status of an animal and only provide information about previous exposure to infection. In contrast to the pCS20 PCR, the serological assay detected the highest proportion of positive animals in the first week of age (0 – 10 days). This was followed by a gradual decline in seropositivity over the 162-day study period with increasing age (Table 2, 3, 4 and Table 6), suggesting widespread presence of maternal antibodies in neonatal lambs and kids born to immune (since they were raised in tick-infested areas exposed to continuous tick challenge) dams maintained under the traditional husbandry system. A similar decline in antibody levels detected by a different serological test was reported over the first 2–3 months of life in traditionally managed calves, lambs and kids in Ghana [47]. The MAP1-B ELISA has high sensitivity for ovine and caprine sera [17,18,20] but is less sensitive for bovine sera, especially in cattle subsequent to the first seroconversion [14,33,47]. However, in the present study, some animals that tested positive by both MAP1-B ELISA and pCS20 PCR later became intermittently negative by MAP1-B ELISA. This suggests the possibility of upregulatory-downregulatory effect on the production of antibodies as reported to occur in cattle during persistent infection [14]. However, persistently high antibody levels have been reported in sheep following immunization [31,48], recovery from experimental heartwater [7], and natural field exposure [21,47]. Thus another possible explanation might be that, if vertical transmission is occurring in small ruminants, the resulting infection in the offspring is atypical in some way and does not result in the persistently high antibody levels seen after experimental or tick-transmitted infection. Immunotolerance to the infectious agent, reported in cattle following pestivirus infection (Bovine Viral Diarrhoea), develops when the virus invades the foetus before development of immune competence resulting in a lifetime of persistence of infection [49]. In this case, the agent is persistently present in the blood without detectable antibodies, which phenomenon, with respect to *E. ruminantium *infection, could be possibly occurring in some extensively managed small ruminants as well; and it could be a mechanism by which vertically *E. ruminantium*-infected offspring survive/tolerate the infection. This requires further investigation. Alternatively, the possibility of vertical transmission resulting in time, in mortality, in immunosuppressed animals due to starvation, *haemonchosis *caused by *Haemonchus contortus *[50] or trypanosomosis [51-53], all of which are common in small ruminants under the traditional husbandry system, should not be entirely discounted. Indeed, mortality due to confirmed cases of heartwater has been observed in sheep after 3–4 weeks of quarantine in tick/insect proof stables prior to use in heartwater vaccination experiments at ITC. These sheep tested *E. ruminantium*-positive by nested pCS20 PCR described above suggesting a carrier status which in time, resulted in mortality possibly due to stress factors such as confinement with inadequate nutrition (authors' unpublished information). Furthermore, in this study, results of the pCS20 PCR and MAP1-B ELISA did not always agree (*r*_*s *_= -0.1512; P = 0.003); similarly the *kappa *statistic determined for the various age categories (Table 7) indicated little agreement between the two assays with agreement ranging between less than is expected to fair, which can be anticipated as both tests target different bio-molecules. For instance, two animals (#2351 and #2352) remained negative throughout the study by pCS20 PCR but tested positive by indirect MAP1-B ELISA; whereas from day 50 – 77 onwards, a number of animals that tested negative by MAP1-B ELISA were positive by pCS20 PCR. On the other hand, a number of animals that tested negative by pCS20 PCR were several times positive by MAP1-B ELISA (Table 2, 3 and 4), which seems to indicate the presence and persistence of maternal antibodies in those animals [47] or that the assay may be detecting antibodies to an uncharacterized closely related ehrlichial organism[19].

This study demonstrated that mortality due to heartwater occurs in young indigenous lambs and kids under a traditional husbandry system in The Gambia as early as 4 weeks after birth. The frequency of mortality was highest in the animals aged between 4 and 12 weeks (Figure 1) suggesting a period of increased risk of/susceptibility to heartwater disease for these animals in this age group. This time interval appears to coincide with the period when the inverse age-related resistance in the newborn has waned. Furthermore, nearly all confirmed cases of heartwater mortality occurred in animals, which tested positive by PCR at 3 weeks of age, or older outside the brief period of inverse age-related resistance suggesting reduced resistance in these animals to any possible latent or new infection. On the other hand, the occurrence of heartwater-associated mortalities in animals prior to week 16, when ticks were first detected, could not be adequately explained. It is postulated that the deaths (8 out of 9) most likely resulted from vertical transmission itself due to possible immunosuppression in the affected animals, or highly unlikely though, that there must have been ticks infesting these animals prior to week 16 which were not detected, and which could have resulted in the positive pCS20 results.

## Conclusion

The findings of this study suggest that both the vector and vertical transmission may play a vital role in the epidemiology of heartwater supporting the view of previous studies [1,9]. Although this would require further investigation, the data presented here, coupled with the traditional system of management of neonatal lambs, kids and their dams in The Gambia seem to support the conclusion that vertical transmission may be crucial in the initial establishment of endemic stability to heartwater in indigenous young sheep and goat population exposed to continuous field tick challenge. The study also supports earlier reports that single-occasion testing of a field-exposed animal may not confirm its actual infection status [29] and the use of pCS20 PCR supported by transmission studies and clinical data would help provide more accurate information on the epidemiology of heartwater (*E. ruminantium *infection) in endemic areas. Additionally, it showed the age range of increased susceptibility to heartwater in traditionally reared small ruminants which information could enhance better targeting of disease control measures especially through vaccination.

## Competing interests

The author(s) declare that they have no competing interests.

## Authors' contributions

BF performed the serology and PCR tests; participated in the coordination and management of the study; contributed to the conception, design, and acquisition of data; did the analysis of data and wrote the manuscript. DG contributed to the conception and design of the study and critical revision of the manuscript; participated in the management of study. SM contributed to the design, coordination and acquisition of data. LBS contributed to interpretation of data and revising the manuscript. FJ contributed to the design and management of the study and revising the manuscript critically for intellectual content. All authors gave their final approval of the version to be published.

## Pre-publication history

The pre-publication history for this paper can be accessed here:



## References

[B1] O´Callaghan CJ, G.F. Medley, T.F. Perry, B.D. Perry (1998). Investigating the epidemiology of heartwater (Cowdria ruminantium infection) by means of a transmission dynamics model. Parasitology.

[B2] Norval RAI, Perry BD, Young AS (1992). Epidemiology of Theileriosis in Africa. Academic Press.

[B3] Perry BD, A.S. Young (1995). The past and future roles of epidemiology and economics in the control of tick-borne diseases of livestock in Africa: The case of theileriosis. Prev Vet Med.

[B4] Uilenberg G (1990). Present and future possibilities for the control of cowdriosis and anaplasmosis. Vet Q.

[B5] Du Plessis JL, Malan L (1987). The non-specific resistance of cattle to heartwater. Onderstepoort J Vet Res.

[B6] Neitz WO, Alexander RA (1941). The immunization of calves against heartwater. J S Afric Vet Med Assoc.

[B7] Uilenberg G (1971). Etudes sur la cowdriose a Madagascar. Premiere partie. Rev Elev Méd Vét Pays Trop.

[B8] Camus E, Barre N (1987). Epidemiology of heartwater in Guadeloupe and in the Caribbean. Onderstepoort J Vet Res.

[B9] Deem SL, R.A.I. Norval, P.L. Donachie, S.M. Mahan (1996). Demonstration of vertical transmission of Cowdria ruminantium, the causative agent of heartwater from cows to their calves. Vet Parasitol.

[B10] Norval RAI, Donachie PL, Meltzer MI, Deem SL, Mahan SM (1995). The relationship between tick (Amblyomma hebraeum) infestation and immunity to heartwater (Cowdria ruminantium infection) in calves in Zimbabwe. Vet Parasitol.

[B11] Waghela SD, Rurangirwa FR, Mahan SM, Yunker CE, Crawford TB, Barbet AF, Burridge MJ, McGuire TC (1991). A cloned DNA probe identifies Cowdria ruminantium in Amblyomma variegatum ticks. J Clin Microbiol.

[B12] Mahan SM, Waghela SD, McGuire TC, Rurangirwa FR, Wassink LA, Barbet AF (1992). A cloned DNA probe for Cowdria ruminantium hybridised with eight heartwater strains and detects infected sheep. J Clin Microbiol.

[B13] Peter TF, Deem SL, Barbet AF, Norval RAI, Simbi BH, Kelly PJ, Mahan SM (1995). Development and evaluation of PCR assay for detection of low levels of Cowdria ruminantium infection in Amblyomma ticks not detected by DNA probe. J Clin Microbiol.

[B14] Semu SM, T.F. Peter, D. Mukwedeya, A.F. Barbet, F. Jongejan, S.M. Mahan (2001). Antibody responses to MAP 1B and other Cowdria ruminantium antigens are down regulated in cattle challenged with tick-transmitted heartwater. Clin Diagn Lab Immunol.

[B15] Du Plessis JL, Bezuidenhout JD, Brett MS, Camus E, Jongejan F, Mahan SM, Martinez D (1993). The sero-diagnosis of heartwater: a comparison of five tests. Rev Elev Méd vét Pays trop.

[B16] Mahan SM, Tebele N, Mukwedeya D, Semu S, Nyathi CB, Wassink LA, Kelly PJ, Peter T, Barbet AF (1993). An immunoblotting assay for heartwater based on the immunodominant 32-kilodalton protein of Cowdria ruminantium detects false positives in field sera. J Clin Microbiol.

[B17] Van Vliet AHM, Van Der Zeijst BAM, Camus E, Mahan SM, Martinez D, Jongejan F (1995). Use of a specific immunogenic region on the Cowdria ruminantium MAP1 protein in a serological assay. J Clin Microbiol.

[B18] Mahan SM, Semu S, Peter T, Jongejan F (1998). Evaluation of MAP1-B ELISA for cowdriosis with field sera from livestock in Zimbabwe. Ann N Y Acad Sci.

[B19] Kakono O, Hove T, Geysen D, Mahan S (2003). Detection of antibodies to the Ehrlichia ruminantium MAP1-B antigen in goat sera from communal land areas of Zimbabwe by an indirect enzyme-linked immunosorbent assay. Onderstepoort J Vet Res.

[B20] De Waal DT, Matthee O, Jongejan F (2000). Evaluation of the MAP1b ELISA for the diagnosis of heartwater in South Africa. Ann N Y Acad Sci.

[B21] Bell-Sakyi L, Koney EBM, Dogbey O, Sumption KJ, Walker AR, Bath A, Jongejan F (2003). Detection by two enzyme-linked immunosorbent assays of antibodies to Ehrlichia ruminantium in field sera collected from sheep and cattle in Ghana. Clin Diagn Lab Immunol.

[B22] Faburay B, Munstermann S, Geysen D, Bell-Sakyi L, Ceesay A, Bodaan C, Jongejan F (2005). Point seroprevalence survey of Ehrlichia ruminantium infection in small ruminants in The Gambia. Clin Diagn Lab Immunol.

[B23] Faburay B, Geysen D, Munstermann S, Taoufik A, Postigo M, Jongejan F (2007). Molecular detection of Ehrlichia ruminantium infection in Amblyomma variegatum ticks in The Gambia. Exp Appl Acarol.

[B24] Faburay B, Munstermann S, Geysen D, Jongejan F (2004). A contribution to the epidemiology of Ehrlichia ruminantium infection (Heartwater) in small ruminants in The Gambia. Animal Health Research Working Paper No. 4, ITC (2004), pp.36.

[B25] Jaitner J, Sowe J, Secka-Njie E, Dempfle L (2001). Ownership pattern and management practices of small ruminants in The Gambia - implications for a breeding programme. Small Rum Res.

[B26] Mattioli RC, Bah M, Reibel R, Jongejan F (2000). Cowdria ruminantium antibodies in acaricide-treated and untreated cattle exposed to Amblyomma variegatum ticks in The Gambia. Exp Appl Acarol.

[B27] Mattioli RC, Janneh L, Corr N, Faye JA, Pandey VS, Verhulst A (1997). Seasonal prevalence of ticks and tick transmitted haemoparasites in traditionally managed N'Dama cattle with reference to strategic tick control in The Gambia. Med Vet Entomol.

[B28] Purchase HS (1945). A simple and rapid method for demonstrating Rickettsia ruminantium (Cowdry, 1925) in heartwater brains. Vet Rec.

[B29] Simbi BH, Mahan SM, T.F. Peter, M.J. Burridge (2003). Comparing the detection of exposure to Ehrlichia ruminantium infection on a heartwater-endemic farm by the pCS20 polymerase chain reaction and an indirect MAP1-B enzyme linked immunosorbent assay. Onderstepoort J Vet Res.

[B30] Geysen D, Delespaux V, Geerts S (2003). PCR-RFLP using Ssu-rDNA amplification as an easy method for species-specific diagnosis of Trypanosoma species in cattle. Vet Parasitol.

[B31] Mboloi MM, Bekker CPJ, Kruitwagen C, Greiner M, Jongejan F (1999). Validation of the indirect MAP1-B enzyme-linked immunosorbent assay for diagnosis of experimental Cowdria ruminantium infection in small ruminants. Clin Diagn Lab Immunol.

[B32] Van Vliet AHM, Van der Zeijst BAM, Camus E, Mahan SM, Martinez D, Jongejan F (1996). Recombinant expression and use in serology of a specific fragment from Cowdria ruminantium MAP1 protein. Ann N Y Acad Sci.

[B33] Peter TF, Mahan SM, C.J. O´Callaghan, G.F. Medley, B.D. Perry, S.M. Semu (2001). Population-based evaluation of the Ehrlichia ruminantium MAP-1B indirect ELISA. Exp Appl Acarol.

[B34] Mondry R, Martinez D, Camus E, Liebisch A, Katz JB, Dewald R, Van Vliet AHM, Jongejan F (1998). Validation and comparison of three enzyme-linked immunosorbent assay for the detection of antibodies to Cowdria ruminantium infection. Ann N Y Acad Sci.

[B35] Jongejan F, Uilenberg G, Franssen FFJ, Gueye A, Nieuwenhuijs J (1988). Antigenic differences between stocks of Cowdria ruminantium. Res Vet Sci.

[B36] Everitt RS (1989). Statistical methods for medical investigations. Oxford Uinversity Press, New York/Edward Arnold, London.

[B37] Wilson JC, Foggie A, Carmichael MA (1964). Tick-borne fever as a cause of abortion and stillbirths in cattle. Vet Rec.

[B38] Dawson JE, Ristic M, Holland CJ, Whitlock RH, Sessions J (1987). Isolation of Ehrlichia risticii, the causative agent of Potomac horse fever, from the fetus of an experimentally infected mare. Vet Rec.

[B39] Zaugg JL (1985). Bovine anaplasmosis: Transplacental transmission as it relates to stage of gestation. Am J Vet Res.

[B40] Fiset P, Wisseman CL, El Bataivi Y (1975). Immunologic evidence of human fetal infection with Coxiella burnetti. Am J Epidemiol.

[B41] Bezuidenhout JD (1988). Certain aspects of the transmission of heartwater, the occurrence of the organims in ticks and in vitro culture. DVSc Thesis, University of Pretoria, South Africa.

[B42] Lawrence JA, Foggin CM, Norval RAI (1980). The effect of war on the control of diseases in livestock in Rhodesia (Zimbabwe). Vet Rec.

[B43] Norval RAI (1983). Arguments against intensive dipping. Zimbabwe Vet J.

[B44] Bezuidenhout JD (1985). The epidemiology and control of heartwater and other tick-borne diseases of cattle in South Africa. Onderstepoort J Vet Res.

[B45] Jongejan F, Uilenberg G (2004). The global importance of ticks. Parasitology.

[B46] Allsopp METP, Van Strijp MF, Farber E, Josemans AI, Allsop BA (2007). Ehrlichia ruminantium  variants which do not cause heartwater found in South Africa. Vet Microbiol.

[B47] Bell-Sakyi L, Koney EBM, Dogbey O, Walker AR (2004). Ehrlichia ruminantium seroprevalence in domestic ruminants in Ghana. I. Longitudinal survey in the Greater Accra Region. Vet Microbiol.

[B48] Du Plessis JL (1981). The application of the indirect fluorescent antibody test to the serology of heartwater. In Tick Biology and Control. Edited by Whitehead GB, Gibson JD. Tick Research Unit, Grahamstown, South Africa.

[B49] Quinn PJ, Markey BK, Carter ME, Donnelly WJ, Leonard FC (2002). Veterinary Microbiology and Microbial Disease. Blackwell Science Ltd. 9600 Garsington Road, Oxford OX4 2DQ, UK. pp 535.

[B50] Osaer S, Goossens B (1999). Trypanotolerance in Djallonke sheep and West African Dwarf goats in The Gambia: Importance of trypanosomosis, nutrition, helminth infections and management factors. PhD Thesis, Utrecht University, Utrecht, The Netherlands.

[B51] Whitelaw DD, Scott JM, Reid HW, Holmes PH, Jennings FW, Urquhart GM (1979). Immunosuppression in bovine trypanosomiasis: studies with loupin-ill vaccine. Res Vet Sci.

[B52] Mattioli RC, Faye JA, Bah M, Jabang B (1994). Experimental Trypanosoma congolense infection on naturally occurring ticks in N'Dama and Gobra zebu cattle. Parasitologia.

[B53] Mattioli RC, Pandey VS, Murray M, Fitzpatrick JL (2000). Immunogenetic influences on tick resistance in African cattle with particular reference to trypanotolerant N'Dama (Bos taurus) and trypanosusceptible Gobra zebu (Bos indicus) cattle. Acta Tropica.

